# Characterization of PVC/CaCO_3_ Nanocomposites Aged Under the Combined Effects of Temperature and UV-Radiation

**DOI:** 10.3390/ma18174001

**Published:** 2025-08-27

**Authors:** Soraya Nait Larbi, Mustapha Moudoud, Abdallah Hedir, Omar Lamrous, Ali Durmus, David Clark, Ferhat Slimani

**Affiliations:** 1 Laboratoire des Technologies Avancées en Génie Electrique, LATAGE, Université Mouloud Mammeri de Tizi Ouzou, BP 17 RP, Tizi-Ouzou 15000, Algeria; soraya.naitlarbi@ummto.dz (S.N.L.); mustapha.moudoud@ummto.dz (M.M.); ferhat.slimani@ummto.dz (F.S.); 2 Advanced High Voltage Engineering Centre, School of Engineering, Cardiff University, Queen’s Buildings, The Parade, Cardiff CF24 3AA, UK; 3 Laboratoire de Physique et Chimie Quantique (LPCQ), Université Mouloud Mammeri de Tizi-Ouzou, BP 17 RP, Tizi-Ouzou 15000, Algeria; omar.lamrous@ummto.dz; 4 Department of Chemical Engineering, Engineering Faculty, Istanbul University-Cerrahpasa, Istanbul 34320, Turkey; durmus@iuc.edu.tr

**Keywords:** calcium carbonate (CaCO_3_), polyvinyl chloride (PVC), aging, dielectric properties, Fourier-transform infrared (FTIR), X-ray spectroscopy (SEM-EDX)

## Abstract

This article examines the influence of micro- and nanoscale calcium carbonate (CaCO_3_) fillers on the dielectric behavior and aging resistance of polyvinyl chloride (PVC)-based composites. PVC films containing varying CaCO_3_ contents (0%, 2.5%, 5%, and 7.5% by weight) were subjected to accelerated aging through prolonged ultraviolet (UV) exposure and thermal stress for up to 1248 h. The evolution of dielectric properties was characterized by impedance spectroscopy, while structural modifications were analyzed using Fourier-transform infrared (FTIR) spectroscopy. Additionally, changes in surface morphology, internal homogeneity (related to particle size, shape, and distribution), and chemical composition were investigated using scanning electron microscopy combined with energy-dispersive X-ray spectroscopy (SEM-EDX), to evaluate the effects of irradiation and variations in the material’s surface composition and morphology. The results reveal a significant correlation between filler concentration and dielectric stability, highlighting the potential of CaCO_3_ reinforcement to improve the long-term reliability of polymeric insulating materials. The results further highlight that beyond the amount of filler used, the fine-scale feature of CaCO_3_, particularly its particle size and how well it is dispersed, has a significant impact on how the material responds to aging and maintains its dielectric properties.

## 1. Introduction

Polyvinyl chloride (PVC) is one of the most widely used thermoplastic polymers, prized for its low cost, excellent chemical and corrosion resistance, good mechanical strength, flexibility, thermal stability, and inherent flame retardancy. These properties make PVC a key material for a wide range of industrial applications, including piping systems, window and door profiles, construction materials, coatings, and more notably, as a dielectric material for medium- and high-voltage electrical components [1,2,3].

When exposed to outdoor environments, PVC faces multiple stress factors such as thermal cycling, mechanical loading, electric fields, ultraviolet (UV) radiation, and corona discharge. The synergy of these factors significantly accelerates aging processes, progressively degrading the material’s physical, chemical, and dielectric properties. This deterioration may ultimately compromise insulating performance, increasing the likelihood of partial discharges or dielectric failure, and substantially reducing service life and recyclability [4,5,6].

Among these stressors, the combined effects of UV radiation and elevated temperatures are particularly detrimental. They induce photodegradation, disrupt the polymer’s microstructure, and impair long-term performance [7,8,9,10]. To address these limitations, considerable research has focused on the incorporation of micro- and nanoscale inorganic fillers into polymer matrices, aiming to enhance their mechanical, thermal, and dielectric properties. Proper dispersion of these fillers enables the efficient absorption and dissipation of UV energy, mitigating structural damage and preserving performance [11,12,13,14].

Reinforced polymer dielectric composites (RPDCs) have thus emerged as promising materials for advanced applications in defense, medical devices, energy systems, and high-performance sports equipment. Their improved dielectric strength, thermal endurance, and mechanical robustness offer significant advantages over conventional insulating materials, especially in demanding outdoor conditions [15,16]. According to the literature, it is well established that the introduction of nanofillers into the PVC matrix improves its properties [17]. Sugumaran [18] reported that SiO_2_ and CaCO_3_ enhance the mechanical and electrical properties of PVC. On the other hand, Croitoru et al. [19] demonstrated that the addition of CaCO_3_ into a PVC matrix can improve the hardness and rigidity of base PVC. Faiza et al. [20] showed that adding ZnO particles improves the hydrophobic property of the base PVC. Xie et al. [21] showed that PVC/ZnO provides stability against environmental stresses.

Currently, calcium carbonate (CaCO_3_) is widely used as a filler in PVC and other polymers [22], mainly because it is inexpensive, easy to obtain, and blends well with the host material. When incorporated in fine particle sizes, especially at the micro or nanoscale, it can influence the material’s performance in several ways. Its presence tends to improve electrical insulation by adjusting the dielectric constant and limiting energy losses [23]. CaCO_3_ can also reduce the rate at which PVC breaks down under exposure to heat and sunlight [24]. It is also shown that adding CaCO_3_ enhances the mechanical performance of PVC [25]. These changes help slow common aging processes, such as oxidation and molecular degradation. By reinforcing PVC in this way, CaCO_3_ contributes to a longer material life and more stable performance in outdoor applications [26]. The effectiveness of CaCO_3_ reinforcement, however, remains highly dependent on particle size, dispersion quality, and filler loading [26,27,28].

This study investigates how varying concentrations of calcium carbonate (CaCO_3_) influence the long-term electrical performance and structural stability of PVC composites exposed to combined thermal and ultraviolet (UV) aging. Attention is given to three main aspects: changes in dielectric properties such as permittivity and dielectric losses—across different frequencies and exposure times; chemical alterations within the polymer matrix identified through FTIR spectroscopy; and surface degradation patterns analyzed using SEM-EDX. The goal is to determine how filler content affects the material’s resistance to environmental stressors, with the broader objective of enhancing the reliability of PVC-based insulation materials used in demanding outdoor and high-voltage applications. Although previous studies, such as the work by Hai et al. [29], have examined the influence of UV exposure on PVC/CaCO_3_ systems, the present study introduces several important distinctions. In this case, the samples were subjected to both ultraviolet radiation and elevated temperatures at the same time, offering a more realistic simulation of outdoor aging conditions. The total aging period was also extended to 1248 h, which allows for a deeper assessment of long-term material behavior. Additionally, special attention was given to the characteristics of the CaCO_3_ filler, particularly its fine particle size, which is a factor that has not been thoroughly addressed in earlier work.

## 2. Experimental Setup

The samples analyzed in this study, prepared by the ENICAB laboratory (Entreprise Nationale de l’Industrie du Cable, Biskra, Algeria), are primarily composed of a standard 4000 M (PVC) matrix resin specifically formulated for wire and cable insulation applications, which is a commercial product manufactured by the National Petrochemical Industries Company (ENIP) in Skikda, Algeria. The material, PVC 4000M, is a thermoplastic polymer produced through a suspension polymerization process. The resin was mixed with CaCO_3_ (anhydrous substance from Sigma-Aldrich, St. Louis, MO, USA, reference 12010-1KG-R, Pcode: 102243766) at proportions of 2.5%, 5%, and 7.5%. The calcium carbonate powder used in this work was selected based on its consistent quality and suitability for polymer processing. With particles averaging no more than 50 μm in size with a purity of about 99%, it enables a relatively uniform distribution in the PVC matrix, which can influence the material’s electrical and aging behavior through interfacial effects. The relative permittivity of PVC is approximately 3.1, while that of CaCO_3_ is 6.1.

The PVC/CaCO_3_ was obtained using a calendering machine, type Polymix 200 P (Brabender, Duisburg, Germany). The machine is equipped with two visible rollers that laminate the polymer. Their temperature can be adjusted independently using the controllers, and in our case, the temperature was set at 170 °C. The heated rollers ensure the polymer material melts. In our case, the preparation of the material was carried out at a processing temperature of 165 °C by mixing the PVC and CaCO_3_ in the desired dosage and introducing them together onto the heated rollers for about 15 min. The samples measured 50 mm × 50 mm × 1.4 mm.

**Figure 1:**
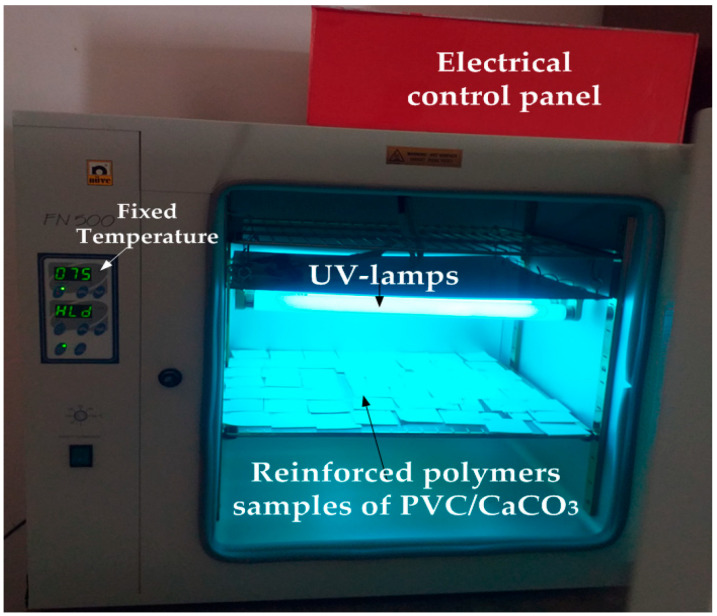
NUVE FN500 chamber used for simultaneous ultraviolet (UV) and thermal aging.

The samples were subjected to simultaneous ultraviolet (UV) radiation and thermal aging, using an experimental ventilated oven of the brand NUVE, model FN500 (NUVE, Ankara, Turkey) (Figure 1). This oven is equipped with six equidistant 15 W Phillips lamps (model TL 15W/10 UV-A, manufactured by Philips Lighting, Netherlands), mounted in parallel. The spectrum of the sources is primarily located in the (UV-A) range, with emitted wavelengths between 350 nm and 400 nm. The lamps also emit a small amount of radiation in the (UV-B) range. The PVC/CaCO_3_ samples were positioned 10 cm away from the UV radiation sources. The aging process was accelerated over a total period of 1248 h (52 days), with cycles of 96 h at 80 °C in a dry heat oven. Only the upper side of the samples was directly exposed to the UV radiation. The exposure intervals were applied as 672 and 1248 h for physicochemical characterizations. Dielectric measurements were performed with an exposure interval of 96 h. After the aging process, the samples were dried in a ventilated oven at 60 °C for 24 h to remove any moisture and ensure the samples were stabilized before performing the physicochemical and dielectric measurements. The aging tests were performed under dry heat conditions in a ventilated oven without humidity control. The influence of moisture, therefore, was not considered in this study, which looked specifically at the combined effects of UV exposure and elevated temperature on the aging behaviour of the material.

The permittivities were measured using a high-precision LCR meter, type Instek-LCR 817 (manufactured by GW Instek, New Taipei City, Taiwan), over the 10 Hz–10 kHz frequency range for all samples, with the real and imaginary parts of the permittivity extracted via a simplified equivalent circuit model that accounted for the sample dimensions. Two measurement campaigns were carried out to reduce the experiments errors, in addition to the high precision LCR-meter of 0.05%.

It is worth recalling that permittivity is a complex quantity that describes the dielectric behavior of materials under various external excitations. The real part of the permittivity, known as the relative permittivity, reflects the material’s ability to store electrical energy through polarization mechanisms and is linked to reactive power. The imaginary part reflects the material’s energy losses specifically due to dielectric and conductive effects, indicating how electrical energy is absorbed and converted into heat within the material. The ratio of the imaginary to the real part of permittivity defines the loss tangent (tan δ), which quantifies the dielectric losses.

The dielectric properties, measured as a function of the applied field frequency, are closely correlated with the aging mechanisms induced by ultraviolet (UV) radiation and thermal exposure. Accordingly, the real and imaginary components of permittivity, along with the dissipation factor (dielectric loss tangent), are determined at each frequency using the following relations [30,31,32,33]:

(1) $$\varepsilon_{r}\left(\omega ,T\right)=\varepsilon_{r}^{\prime }\left(\omega ,T\right)-j\varepsilon_{r}^{\prime \prime }\left(\omega ,T\right)$$

(2) $$\tan\left[\delta \left(\omega ,T\right)\right]=\frac{\varepsilon_{r}^{\prime \prime }\left(\omega ,T\right)}{\varepsilon_{r}^{\prime }\left(\omega ,T\right)}+\frac{\sigma_{dc}}{\omega \varepsilon_{o}\varepsilon_{r}^{\prime }\left(\omega ,T\right)}=\frac{\varepsilon_{r}^{\prime \prime }\left(\omega ,T\right)+\frac{\sigma_{dc}}{\omega \varepsilon_{o}}}{\varepsilon_{r}^{\prime }\left(\omega ,T\right)}$$

**Figure 2:**
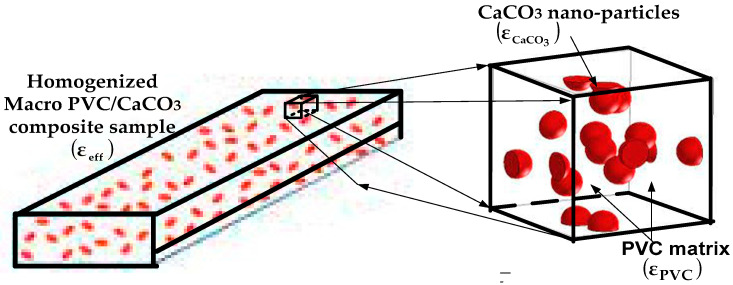
Schematic of PVC/CaCO_3_ composite material [33,34,35].

This study focuses on a homogeneous dielectric composite consisting of polyvinyl chloride (PVC) as the matrix and calcium carbonate (CaCO_3_) as the dispersed filler phase, as illustrated in Figure 2. The PVC matrix has a permittivity ε-PVC, while the CaCO_3_ inclusions are characterized by ε-CaCO_3_. The effective complex permittivity of such polymer nanocomposites depends on several factors, including microstructure, filler volume fraction, inclusion geometry and distribution, and the nature of interfacial interactions between matrix and filler.

Although various analytical and numerical models can provide useful estimates of the effective permittivity in two-phase systems, experimental characterization remains essential, particularly for materials with non-ideal or disordered structures.

Several theoretical models have been developed to predict the effective dielectric properties of two-phase systems such as polymer/ceramic composites. A commonly used approach is the logarithmic mixing rule [34], which expresses the effective permittivity as a function of the component permittivities and volume fractions. Other models introduce corrections to better account for filler morphology and interfacial effects [33]. Frequency-dependent behavior has also been modeled using extended mixing rules incorporating dielectric dispersion mechanisms [35]. Additionally, advanced models consider not only the intrinsic properties of matrices and fillers, but also the geometric aspects of inclusions to predict the effective dielectric response [35]. In this context, the homogenized dielectric properties of the composite can be expressed by the following relation:

(3) $$\varepsilon_{eff}\left(\omega ,T\right)=f{\left(\varepsilon_{pvc}\left(\omega ,T\right)\right)}^{\beta }+\left(1-f\right){\left(\varepsilon_{CaCO_{3}}\left(\omega ,T\right)\right)}^{\beta }$$

Here, *f* represents the volume fraction of the PVC matrix, (1 − *f*) corresponds to the filler phase and *β* is a fitting parameter which controls how the dielectric properties of the individual components are mixed. The dissipation factor is estimated as follows:

(4) $$tan\left(\delta_{eff}\left(\omega ,T\right)\right)=f\cdot tan\left(\delta_{pvc}\left(\omega ,T\right)\right)+\left(1-f\right)tan\left(\delta_{CaCO_{3}}\left(\omega ,T\right)\right)$$

Moreover, the electrical conductivity of the composite is determined as follows:

(5) $$\sigma =\left(\varepsilon_{eff}\left(\omega ,T\right)\right)\omega \cdot tan\left(\delta_{eff}\left(\omega ,T\right)\right)$$

Infrared spectra were also recorded using a SHIMADZU IRAffinity-1S instrument (Shimadzu Corporation, Kyoto, Japan), operating at a resolution of 4 cm^−1^ across the 4000 to 500 cm^−1^ range, and curve fitting was performed to identify the characteristic bands of unaged virgin PVC as well as those of PVC/CaCO_3_ samples doped with 2.5%, 5%, and 7.5% after UV–thermal aging. SEM-EDX analyses were carried out using a Talos F200X scanning electron microscope (manufactured by Thermo Scientific, Waltham, MA, USA) operated in high vacuum mode with an ETD secondary electron detector, at an accelerating voltage of 10 kV.

## 3. Results and Discussion

**Figure 3:**
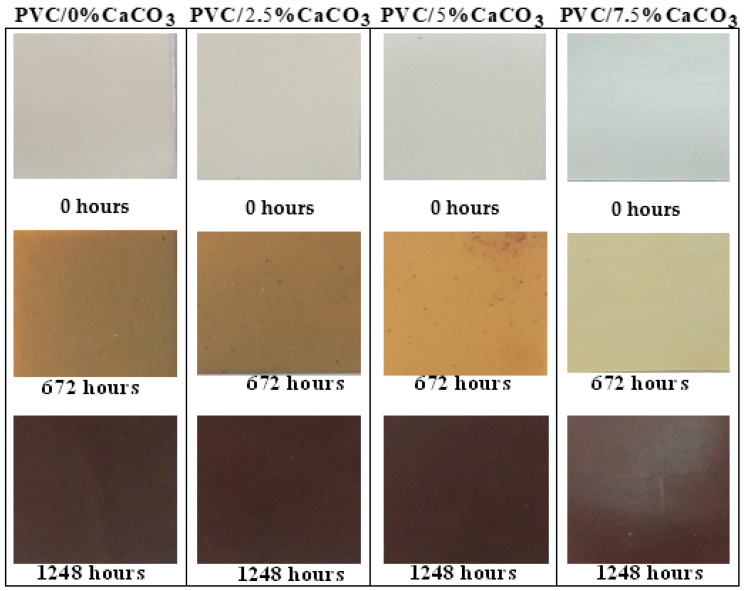
UV–thermal aging effects on the texture and coloration of virgin and PVC/CaCO_3_ composites at different filler contents.

Figure 3 illustrates the changes in texture and color of the samples subjected to simultaneous UV and thermal stresses. For virgin PVC, increased exposure time typically observed after approximately 672 h would lead to a gradual darkening of color and a loss of transparency. These alterations became less pronounced as the doping percentage increased, as has also been reported in [36]. The observed discoloration could reflect oxidation phenomena induced by UV radiation, as prolonged exposure may lead to an increased absorption of high-energy photons by the PVC, thereby enhancing chain scission mechanisms and promoting the formation of chromophore groups.

Although polymers exhibit a degree of stability at ambient temperature, higher temperatures would intensify texture and color changes. At elevated temperatures, the efficiency of heat transfer would increase, enhancing the mobility and penetration of oxygen molecules and thereby accelerating degradation reactions, as proposed in [36,37,38,39,40]. Photo-oxidation, resulting from molecular excitation, would also cause surface hardening, which could lead to cracking and chain fragmentation. These findings could suggest that simultaneous UV and thermal stresses accelerate the oxidation rate, thereby initiating the photodissociation of the macromolecules responsible for the degradation of the dielectric and mechanical properties of the PVC/CaCO_3_ composite [29,41].

Furthermore, UV-induced aging of PVC/CaCO_3_ nanocomposites evaluated through whiteness or UV-absorption measurements indicates that CaCO_3_ may significantly enhance the anti-aging properties of the polymer when optimally doped. The degradation rate would depend on the radiation’s intensity and wavelength, as well as on operating temperature factors that vary with season, latitude, altitude, and exposure duration.

**Figure 4:**
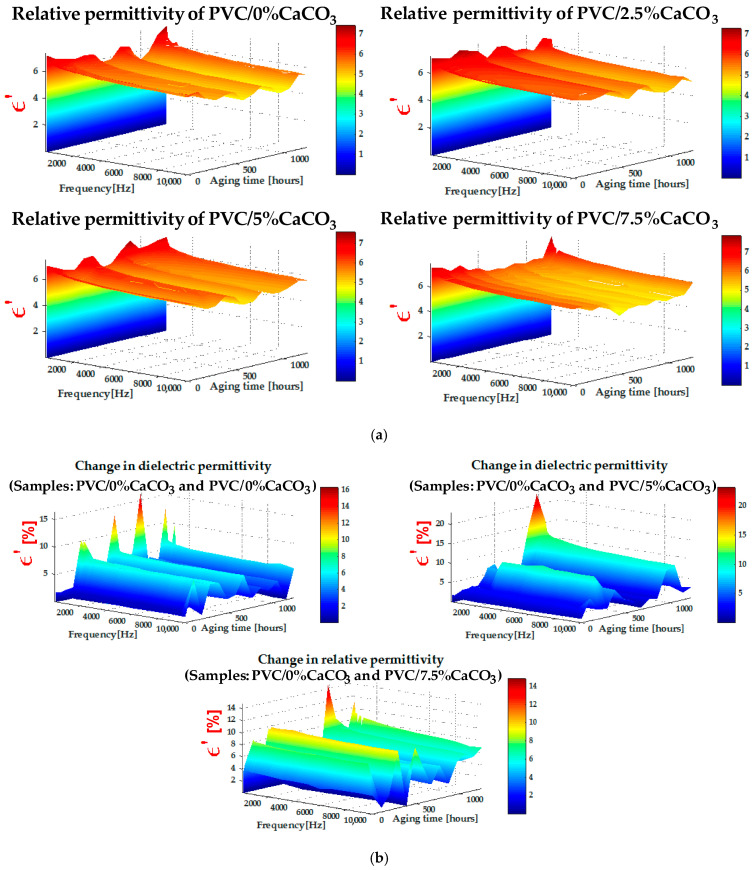
(**a**) Relative permittivity (z-axis) versus aging time (y-axis) and frequency (x-axis) dependence, and (**b**) change of relative permittivity (z-axis) versus aging time (y-axis) and frequency (x-axis) dependence.

Figure 4a shows the evolution of the real part ε′ of the relative permittivity as a function of frequency for different CaCO_3_ doping levels. In general, ε′ decreases with increasing frequency, which is typical of polymer-based materials due to the reduced ability of dipoles to follow the alternating electric field. However, at low frequencies, a slight increase in ε′ may occur, attributable to Maxwell–Wagner–Sillars-type interfacial polarization, arising from the interfaces between the polymer matrix and the CaCO_3_ particles. Increasing the CaCO_3_ content modifies this behavior: a slight initial decrease in ε′ is observed at low filler concentrations (2.5% and 5%), followed by stabilization or even a moderate increase during UV–thermal aging. This evolution can be attributed to the progressive interactions between CaCO_3_ nanoparticles and the PVC polymer chains, and to the formation of polar nanodielectric regions that promote controlled interfacial polarization. At the same time, UV–thermal aging accelerates the degradation of dielectric properties through oxidative reactions and moisture absorption, as reported in various studies [42,43,44,45,46,47].

**Figure 5:**
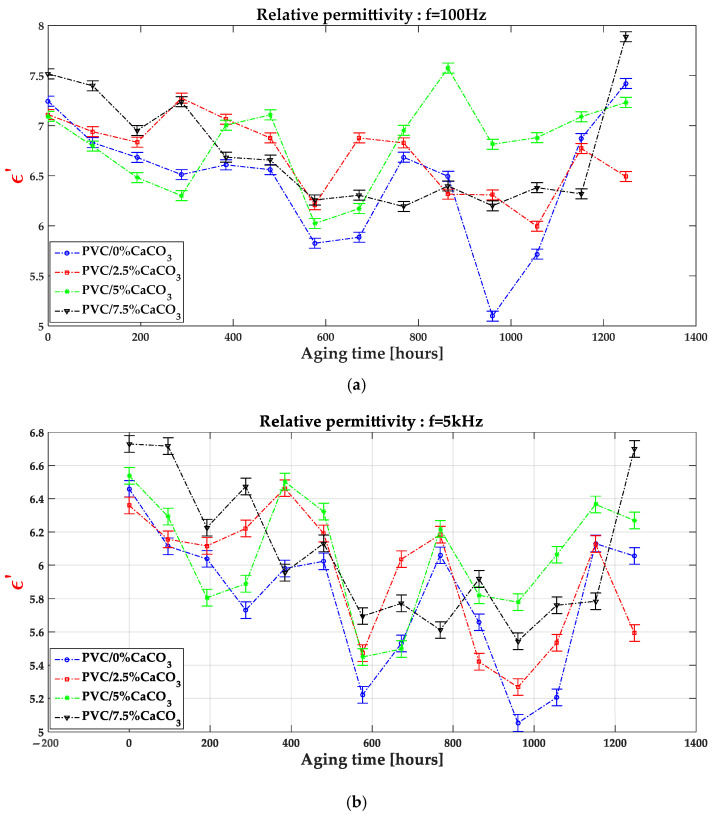

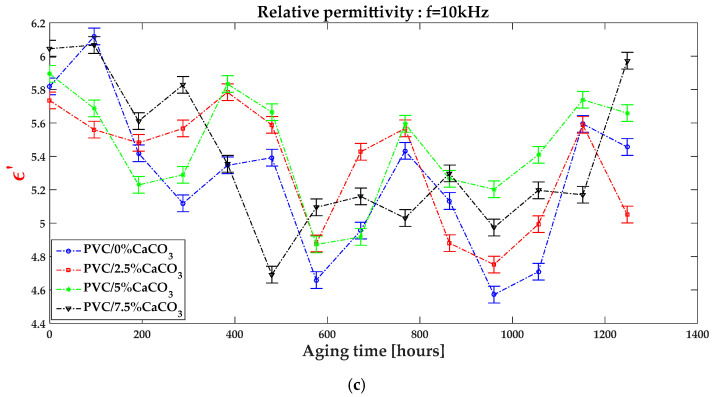
Relative permittivity as function of aging time and CaCO_3_ doping level at different frequencies f = 100 Hz (**a**), f = 5 kHz (**b**) and f = 10 kHz (**c**).

Nevertheless, the presence of CaCO_3_ at optimal concentrations contributes to protection against degradation, limiting the mobility of degraded chains and contributing to the dielectric stabilization of the composite. The formation of such polar regions is strongly influenced by the dispersion quality of the CaCO_3_ particles within the PVC matrix. The SEM observations, reported in the following section, confirm this good dispersion. These localized polar regions contribute to interfacial polarization, while reducing dielectric losses and improving breakdown strength, as further illustrated in Figure 5. The incorporation of nanofillers into polymer dielectrics can therefore enhance permittivity, reduce dielectric losses, and improve breakdown behavior, positioning these nanostructured composites as highly promising materials for high-voltage and power electronics applications. The change of the real part of relative permittivity depicted by Figure 4b exhibits increased values which show the positive impact of the CaCO_3_ on maintaining the good dielectric properties of the doped PVC.

**Figure 6:**
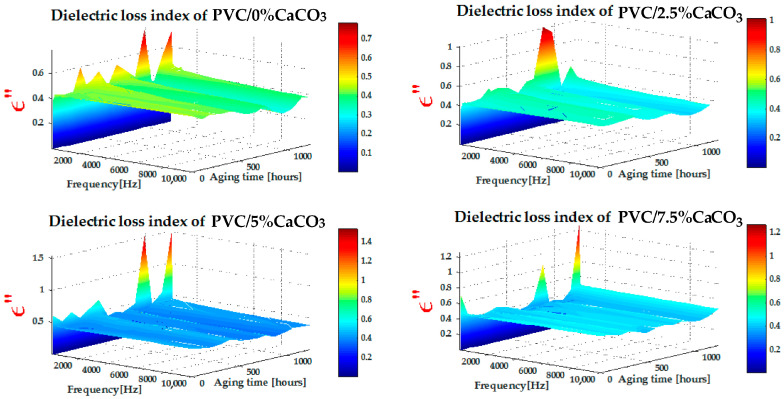
Dielectric loss index of PVC and CaCO_3_ doping level as function of frequency (x-axis) and UV–thermal aging time (y-axis).

The values of the dielectric loss index presented in Figure 6 were obtained from RLC meter measurements, as a function of frequency and exposure time to combined UV–thermal stresses. Figure 6 shows that the dielectric loss index exhibits relatively higher values at low frequencies ([10 Hz–300 Hz]), decreasing progressively as the frequency increases in the range of [500 Hz–10 kHz]. At low frequencies, during the polarization process, the movement and alignment of electric dipoles contribute to an increase in dielectric loss. In contrast, at higher frequencies, relaxation phenomena dominate, leading to a decrease in the dielectric loss index. In addition, the dielectric loss values of the PVC/CaCO_3_ nanocomposites are significantly lower than those of pure PVC, with the effect becoming more pronounced as the CaCO_3_ doping level increases and with prolonged exposure to UV–thermal stresses. The gradient of the dielectric loss index with respect to frequency and aging time decreases as the CaCO_3_ content increases, indicating an improvement in dielectric stability. This behaviour can be attributed to the ability of CaCO_3_ particles to inhibit molecular mobility and trap charge carriers, thereby reducing polarization losses. These trends are consistent with previous studies reported in the literature [29,40,41,42].

**Figure 7:**
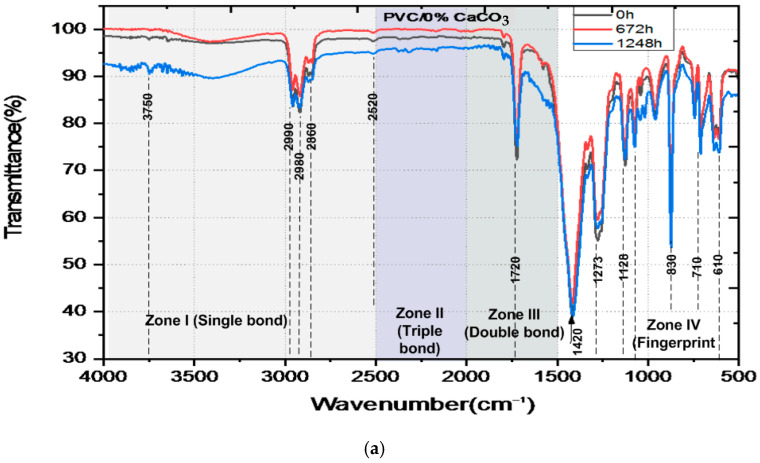

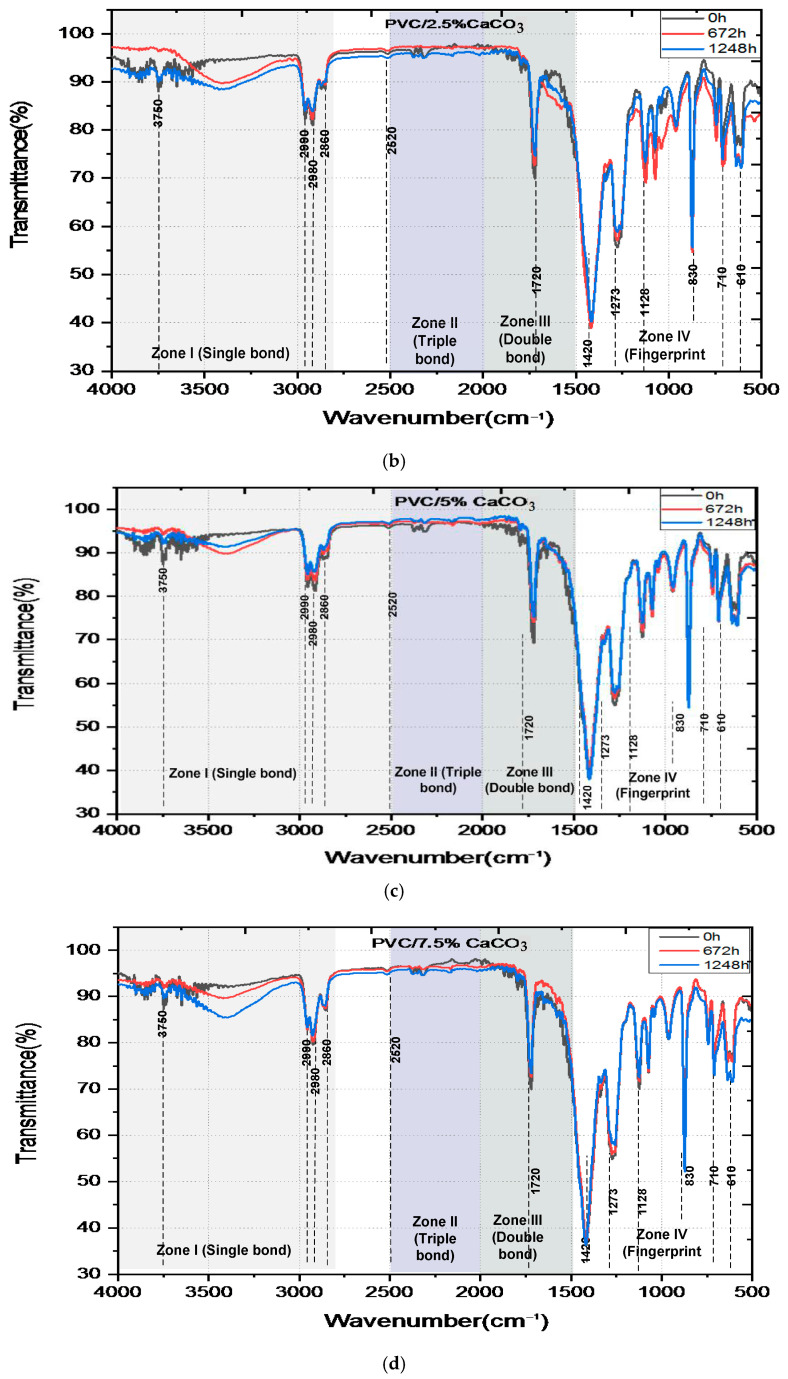

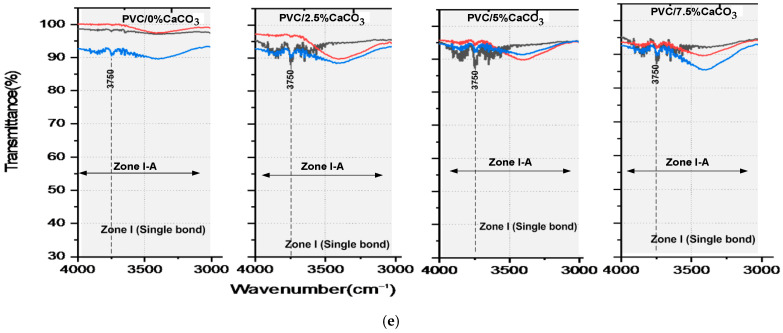
FTIR-ATR spectra of unaged and aged pure PVC (**a**) and PVC/CaCO_3_ nanocomposites with 2.5 wt.% (**b**), 5 wt.% (**c**), 7.5 wt.% (**d**) CaCO_3_ content, subjected to simultaneous UV–thermal stresses, and (**e**) emerging bands in the single bond region (black 0 h, red 672 h and blue 1248 h).

The FTIR spectra of pure PVC and PVC/CaCO_3_ nanocomposites subjected to combined UV–thermal aging for 0, 672, and 1248 h are shown in Figure 7. The spectra can be divided into four main regions. The first region wavenumber, from 2500 to 4000 cm^−1^, corresponds to the stretching vibrations of (C–H) and (O–H) bonds. Several peaks were identified, such as the peaks in the area of 2800–3000 cm^−1^ associated with the presence of (C–H) bonds, and the peaks in the area around 3500–3750 cm^−1^ indicating the presence of (O–H) bonds. High transmittance values (>90%) indicate low vibrational energy absorption due to few exciting photons, even after prolonged exposure. The second region wavenumber, from 2000 to 2500 cm^−1^, reveals weak peaks associated with triple bonds (C≡C) involving stretching vibration mode. The small peaks around 2250 cm^−1^ are related to the presence of a (C–H) group. The third region, between 1500 and 2000 cm^−1^, exhibits a prominent absorption band at 1411 cm^−1^, which intensifies with aging time, indicating the formation of (C–C) carbonyl groups with bending molecular vibrations characteristics, according to the increased absorption (decreased transmittance) linked to oxidative degradation. Finally, the region of wavenumbers below 1500 cm^−1^, known as the fingerprint region and used to uniquely identify molecules, includes bending mode from (CH_2_), and stretching vibrations from (C–H), and (C–Cl) bonds, with noticeable peaks around 830, 710, and 610 cm^−1^ The highest peaks around 1500 cm−1 and 850 cm^−1^ are associated with the (C–H_2_) bending vibrations at 1400 cm^−1^, the (C-H) bending vibrations at 1280 cm^−1^ and 1130 cm^−1^, and the (C–H_2_) rocking vibration at 870 cm^−1^ [48,49,50,51,52,53]. A comparison of the spectra highlights a progressive decrease in transmittance with exposure time, which is more pronounced in pure PVC. The incorporation of CaCO_3_ mitigates this trend, demonstrating improved chemical stability. The composite containing 7.5% CaCO_3_ exhibits the highest resistance to UV–thermal degradation.

The data shown in Table 1 indicate how the transmittance near 1410 cm^−1^ (associated with fundamental functional groups like C–O and CH_2_) changes with aging. Both the PVC and the composites containing CaCO_3_ display changes, or trends in values, with aging at 672 h (slight decrease) and at 1248 h (slight increase). Such changes may correlate to slow chemical changes in the plastic due to weathering under UV and heat which can include oxidation. Composites containing CaCO_3_ were consistently transmitted across the same interval compared to pure PVC. The sample made with 7.5% calcium carbonate filler maintained consistent absorbance for the full duration of the aging testing. The consistent values, shown by their transmittance, is an indication that the CaCO_3_ is added to slow the degradation of the chemical polymer chains to some degree during exposure.

**Figure 8:**
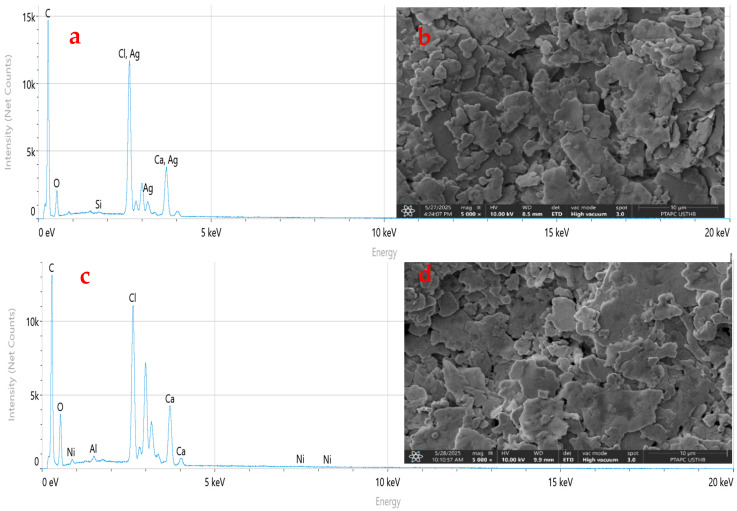
SEM micrographs and EDX spectra of unaged virgin PVC (**a**,**b**) and PVC/CaCO_3_ composite containing 7.5 wt.% CaCO_3_ after 1248 h of UV–thermal aging (**c**,**d**). Images were taken at 5000× magnification with a scale bar of 10 µm.

Figure 8 presents the SEM micrographs and EDX spectra of unaged virgin PVC and PVC/CaCO_3_ composite (7.5 wt.% CaCO_3_) after 1248 h of UV–thermal aging. Figure 8d exhibited no visible alteration in morphology due to aging. This implies the CaCO_3_ particles help to provide structural reinforcement through resistance to surface degradation and the reduction of defect propagation. The better morphology also supports the premise that the filler functions as a physical barrier against UV radiation and thermal stress, thus allowing the material to better retain its integrity during aging. These observations are consistent with the FTIR results (Figure 7), where the progressive formation of carbonyl groups and decrease in transmittance were more pronounced in virgin PVC compared to the composite. The corresponding EDX spectra (Figure 8a,c) confirm the chemical composition of the samples. For virgin PVC, EDX analysis shows C, Cl, and O, with elemental ratios of 70.4%, 7.13%, and 14.9%, respectively. In contrast, the PVC/7.5 wt.% CaCO_3_ composite exhibits C, Cl, O, and Ca, with ratios of 68.52%, 5.62%, 21.18%, and 3.9%, respectively, along with trace amounts of Al (0.6%), Ni (0.12%), Si (0.04%), and Mg (0.02%). These impurities likely arise from the processing and the industrial environment [54,55,56]. The impurities noted can be trace back primarily to the CaCO_3_ powder itself, where organic volatiles and residual solvent species exist in trace amounts (including, e.g., CH_3_COOH- insoluble ≤ 0.2%, HCl-insoluble ≤ 0.002%, and SO_4_^2−^-based alkali or Mg ions ≤ 1%). Further contamination during mixing could also occur through environmental exposure and equipment contact. These impurities can lead to dielectric instability and therefore increase the rate and amount of photodegradation which would arise through charge transport and molecular degradation pathways. These concentrations are below the measurement limit of the EDX method and are therefore negligible. The differences between the presence and levels of this impurity element in samples could be due to differences in interaction with the processing tools, surface exposure for preparing, or small inconsistencies in the dispersion of the fillers throughout the matrix. While these elements are present in small amounts, their presence even at trace levels could influence localized electrical behavior or could accelerate degradation over time, and may have an influence on charge movement or cause a change or reaction in the polymer chemically. In either case, their influence should be considered when reflecting on the aging behavior and dielectric characteristics of the composites. Moreover, the presence of CaCO_3_ appears to inhibit the formation of deep surface defects and contributes to the improved dielectric stability observed previously (Figure 4, Figure 5 and Figure 6), as evidenced by the reduced dielectric loss and more stable permittivity. Overall, the SEM-EDX results support the conclusions drawn from the dielectric and FTIR analyses, highlighting the beneficial role of CaCO_3_ in enhancing the chemical and morphological stability of the PVC-based composites under combined UV–thermal stresses.

## 4. Conclusions

The present study demonstrates that incorporating CaCO_3_ nanoparticles into a PVC matrix leads to significant improvements in the dielectric and chemical stability of the resulting composites under simultaneous UV–thermal aging conditions. The addition of CaCO_3_ reduces dielectric losses, enhances real permittivity, and stabilizes dielectric strength across a wide frequency range. These improvements are closely linked to the filler’s ability to promote interfacial polarization, limit chain scission, and reduce defect formation. FTIR analyses reveal that PVC/CaCO_3_ composites exhibit a slower rate of oxidative degradation and a lower formation of carbonyl groups compared to pure PVC, confirming their enhanced resistance to photodegradation and chemical aging. SEM-EDX observations further support these findings, showing that the composite microstructure remains more homogeneous and less damaged after prolonged exposure, particularly at higher filler contents. Among the tested materials, the PVC/7.5 wt.% CaCO_3_ composite exhibited the best overall performance, combining improved dielectric properties, chemical stability, and morphological integrity. These results suggest that optimized PVC/CaCO_3_ nanocomposites are highly promising candidates for demanding applications such as high-voltage power transmission cables, energy storage devices, and advanced solid-state electronic systems operating in harsh environments. Future work should focus on comprehensive mechanical characterization, long-term aging studies under variable environmental conditions, and detailed modeling of the local dielectric behavior to further tailor these materials for industrial deployment.

## Figures and Tables

**Table 1:** Transmittance around 1410 cm^−1^ for (C-O) and (CH2) groups.

Transmittances (%)	O (around 1410 cm^−1^)			CH_2_ (around 1410 cm^−1^)		
	Aging Time (hours)					
	0 h	672 h	1248 h	0 h	672 h	1248 h
**PVC/0%CaCO_3_**	60	57	63	24	28	30
**PVC/2.5%CaCO_3_**	60	62	60	28	25	28
**PVC/5%CaCO_3_**	65	60	65	27	27	29
**PVC/7.5%CaCO_3_**	75	75	75	27	27	27

## Data Availability

The original contributions presented in this study are included in the article. Further inquiries can be directed to the corresponding authors.
